# Factors involved in ICU mortality following medical retrieval: identifying differences between ICU survivors and nonsurvivors

**DOI:** 10.1186/cc12218

**Published:** 2013-03-19

**Authors:** M Kennedy, P Visser, L Harriss

**Affiliations:** 1 Ambulance Victoria, Melbourne, Australia; 2 Monash University, Melbourne, Australia

## Introduction

The study aimed to determine factors related to ICU mortality in critically ill patients transferred by Adult Retrieval Victoria (ARV) medical staff. Patients who died in the ICU after interhospital transfer were compared with those who survived.

## Methods

This is a retrospective cohort study of ARV cases between 1 January 2009 and 30 June 2010. Retrieval data were linked with data from the ANZICS CORE APD (Australia and New Zealand Intensive Care Society Centre for Outcome and Resource Evaluation Adult Patient Database). Victoria Data Linkage (VDL) performed linkage of the data. Data included demographic and clinical data obtained during transfer and data related to outcome measures in the ICU.

## Results

Of the 601 cases transferred by ARV during the study period, 549 were eligible for linkage to 25,543 ANZICS APD case records for the same period. VDL matched 460 of these cases (83.8%). Logistic regression analysis revealed the variables associated with mortality were advanced age (OR = 1.02, 95% CI = 1.00 to 1.04, *P *= 0.02), cardiac principal referral problems (OR = 1.84, 95% CI = 1.02 to 3.32, *P *= 0.04), lower mean arterial blood pressure (OR = 0.97, 95% CI = 0.95 to 0.99, *P *= 0.005) and tachycardia (OR = 1.02, 95% CI = 1.00 to 1.03, *P *= 0.008) on arrival at the destination hospital.

## Conclusion

Advanced age, lower mean arterial blood pressure and tachycardia towards the completion of transfer were associated with increased ICU mortality in this population. Clinicians should be aware of the additional risk for cardiac patients.
